# Supplementary material for: Protein prenylation restrains innate immunity by inhibiting Rac1 effector interactions
Source: Nat Commun. 2019 Sep 4;10:3975. doi: 10.1038/s41467-019-11606-x (PMC6726657; doi:10.1038/s41467-019-11606-x)
Supplement: Supplementary file 1 — Supplementary Information [file 41467_2019_11606_MOESM1_ESM.pdf]

Supplementary Information

Protein prenylation restrains innate immunity by inhibiting Rac1 effector interactions

Akula et al

Supplementary Figure 1

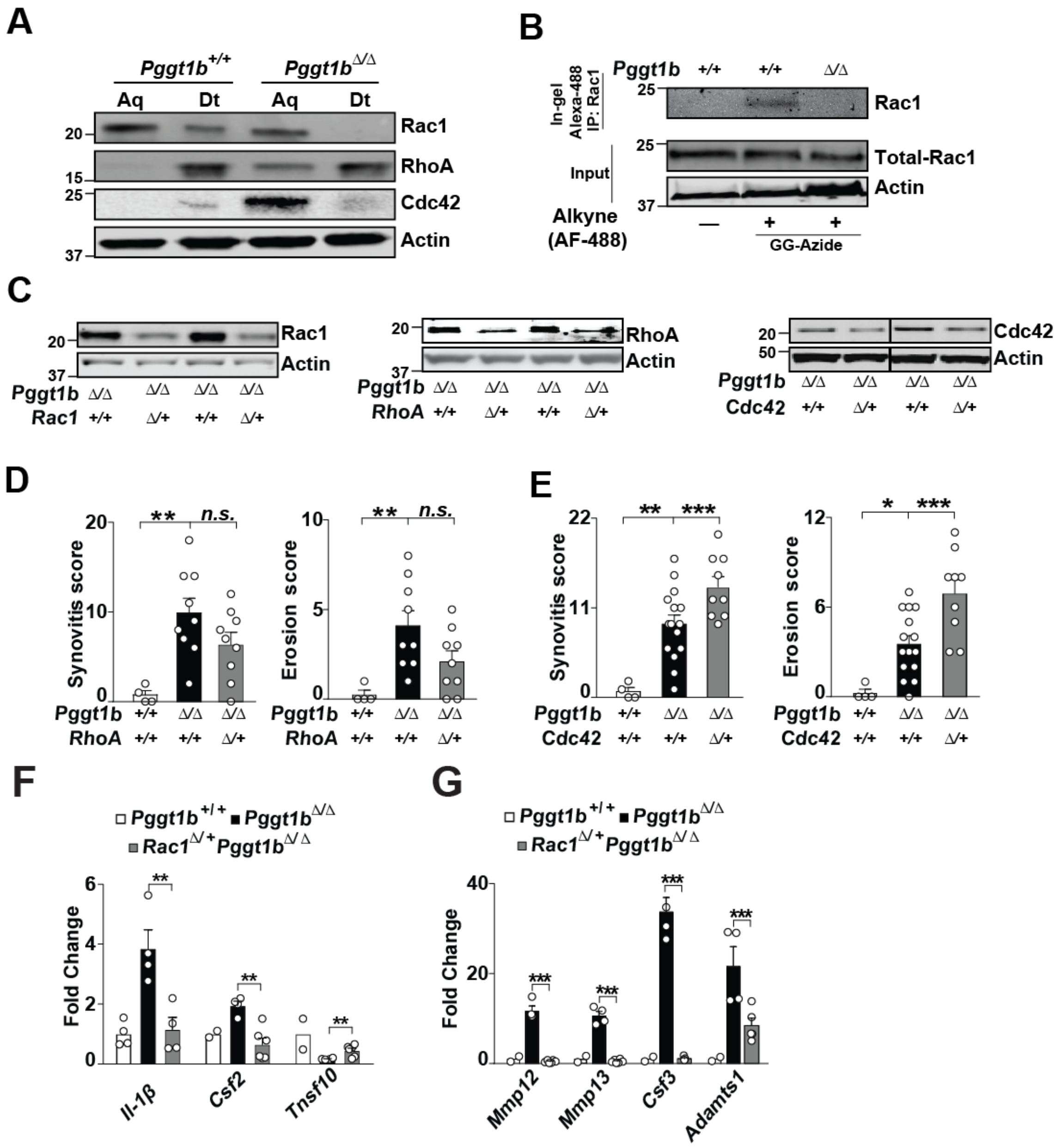

Supplementary Figure 1. Knockout of one copy of *Rac1*, but not *Rhoa*, and *Cdc42*, reduces inflammation-associated phenotypes induced by *Pggt1b*$^{\Delta/\Delta}$ macrophages. (**A**) Left, western blots showing levels of Rac1, RhoA, and Cdc42 in the aqueous and detergent phase in lysates isolated from *Pggt1b*$^{\Delta/+}$ and *Pggt1b*$^{\Delta/\Delta}$ macrophages. (**B**) In-gel fluorescence scan of immunoprecipitated (IP) Rac1 labelled with Alexa Fluor 488 conjugated geranylgeranyl-azide in lysates from macrophages. Twice the amount of total proteins were loaded from the *Pggt1b*$^{\Delta/\Delta}$ samples compared to *Pggt1*$^{+/+}$ to obtain similar total Rac1 levels. Direct western blots were performed on the same lysates (Input) to quantify total Rac1 and Actin. (**C**) Western blots showing levels of Rac1, RhoA, and Cdc42 in lysates of BM macrophages isolated from *Rac1*$^{\Delta/+}$*Pggt1b*$^{\Delta/\Delta}$, *RhoA*$^{\Delta/+}$*Pggt1b*$^{\Delta/\Delta}$, *Cdc42*$^{\Delta/+}$*Pggt1b*$^{\Delta/\Delta}$, and littermate control *Pggt1b*$^{\Delta/\Delta}$ mice. (**D**) Synovitis and erosion scores in joints of 12-week-old *Pggt1b*$^{+/+}$ (n = 4), *Pggt1b*$^{\Delta/\Delta}$ (n = 9) and *RhoA*$^{\Delta/+}$*Pggt1b*$^{\Delta/\Delta}$ (n = 9) mice. (**E**) Synovitis and erosion scores in joints of 12-week-old *Pggt1b*$^{+/+}$ (n = 4), *Pggt1b*$^{\Delta/\Delta}$ (n = 15), and *Cdc42*$^{\Delta/+}$*Pggt1b*$^{\Delta/\Delta}$ (n = 10) mice. (**F, G**) Quantitative polymerase chain reaction (QPCR) of NFκB-regulated (**F**) and extracellular matrix–associated (**G**) genes in cDNA isolated from BM macrophages of *Pggt1b*$^{+/+}$, *Pggt1b*$^{\Delta/\Delta}$, and *Rac1*$^{\Delta/+}$*Pggt1b*$^{\Delta/\Delta}$ mice (n = 3–4/genotype), 8 hours after LPS stimulation. Error bars represent s.e.m. Significance between groups were calculated with two tailed Student's *t* test **(F, G)** and one-way ANOVA with Tukey's post hoc test **(D, E)**. * $P < 0.05$, ** $P < 0.01$, *** $P < 0.001$.

Supplementary Figure 2

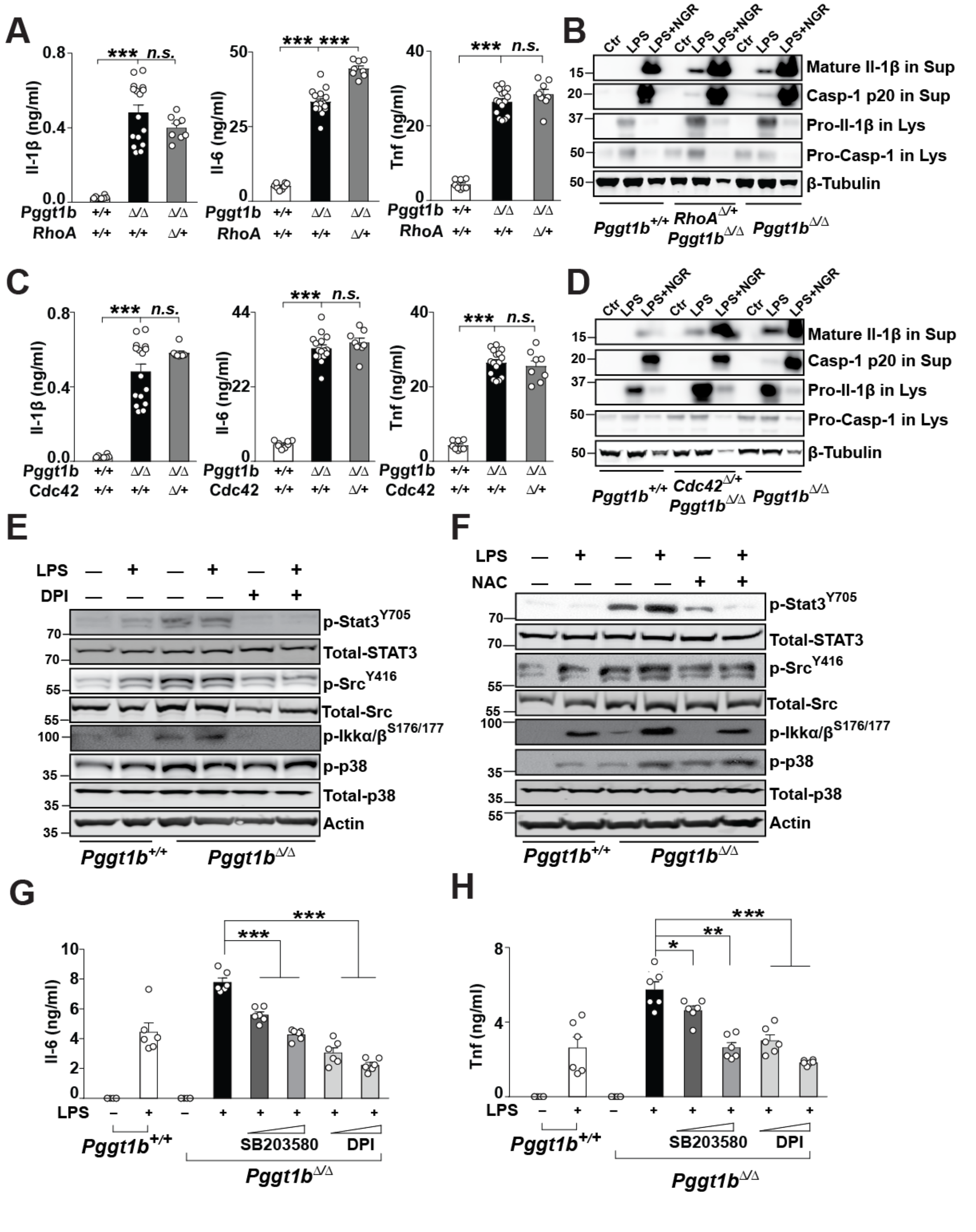

Supplementary Figure 2. The role of RhoA, Cdc42, and intracellular signaling pathways in the increased LPS-stimulated cytokine production in GGTase-I-deficient macrophages. (**A**) Cytokine concentrations, 8 hours after LPS stimulation, in medium of BM macrophages isolated from *Pggt1b*$^{+/+}$, *Pggt1b*$^{\Delta/\Delta}$, and *Rhoa*$^{\Delta/+}$*Pggt1b*$^{\Delta/\Delta}$ mice. (**B**) Western blots showing levels of mature Il-1β and caspase-1 in supernatants (Sup) and pro-Il-1β and pro-caspase-1 in lysates (Lys) of BM macrophages isolated from *Pggt1b*$^{+/+}$, *Pggt1b*$^{\Delta/\Delta}$, and *Rhoa*$^{\Delta/+}$*Pggt1b*$^{\Delta/\Delta}$ mice, 8 hours after LPS stimulation; Tubulin in lysates was used as a loading control. The antibiotic nigericin (NGR) was used as a positive control for inflammasome-mediated caspase-1 activation and Il-1β production. (**C, D**) Similar experiments as in panels **A** and **B** using BM macrophages from *Pggt1b*$^{+/+}$, *Pggt1b*$^{\Delta/\Delta}$, and *Cdc42*$^{\Delta/+}$*Pggt1b*$^{\Delta/\Delta}$ mice. (**E, F**) Western blots showing phosphorylated (p) and total levels of intracellular signaling mediators in lysates of BM macrophages incubated with or without DPI (10 µM) for 10 hours (**E**) and NAC (1 mM) for 16 hours (**F**). (**G, H**) Concentration of Il-6 (**G**) and Tnf (**H**) in medium of LPS-stimulated BM macrophages (n = 3/genotype) that had been pre-incubated for 1 h with inhibitors of p38 (SB203580; 1 and 5 µM) and ROS (DPI; 500 nM and 5 µM). For all panels similar results were observed in two-three independent experiments (n = 3–4/genotype). Error bars represent s.e.m. Significance between groups were calculated with two tailed Student's *t* test. *n.s.* not significant, * $P < 0.05$, ** $P < 0.01$, *** $P < 0.001$.

Supplementary Figure 3

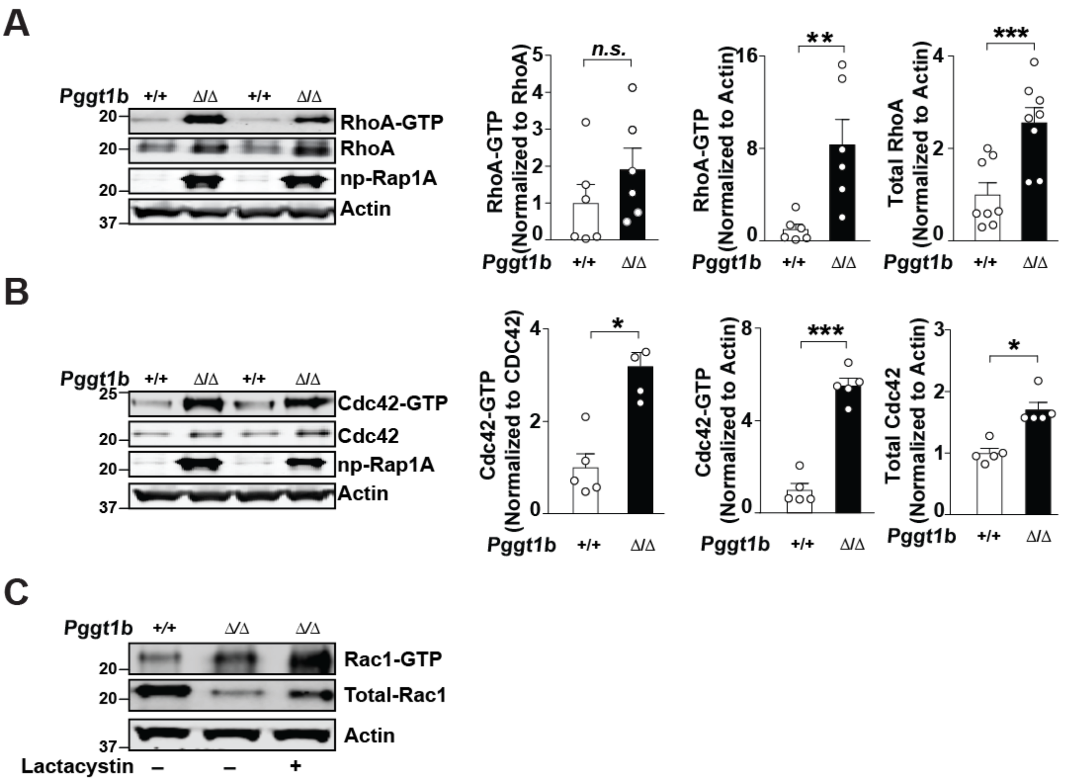

Supplementary Figure 3. Knockout of GGTase-I increases GTP-loading and total levels of RhoA and Cdc42 in macrophages. (**A, B**) Left panels, western blots showing steady-state levels of GTP-bound and total RhoA (**A**) and Cdc42 (**B**) in BM macrophages. Non-prenylated Rap1A was used as marker of GGTase-I-deficient cells; Actin was used as a loading control. Right panels show the amount of GTP-bound and total proteins in BM macrophages (n = 4/genotype) determined by densitometry. (**C**) Western blots showing steady-state levels of GTP-bound Rac1 and total Rac1 in *Pggt1b*$^{+/+}$ cells and in *Pggt1b*$^{\Delta/\Delta}$ cells incubated with lactacystin (10 µM) for 10 hours. Error bars represent s.e.m. Significance between groups were calculated with two tailed Student's t test. *n.s.* not significant, * $P < 0.05$, ** $P < 0.01$, *** $P < 0.001$.

Supplementary Figure 4

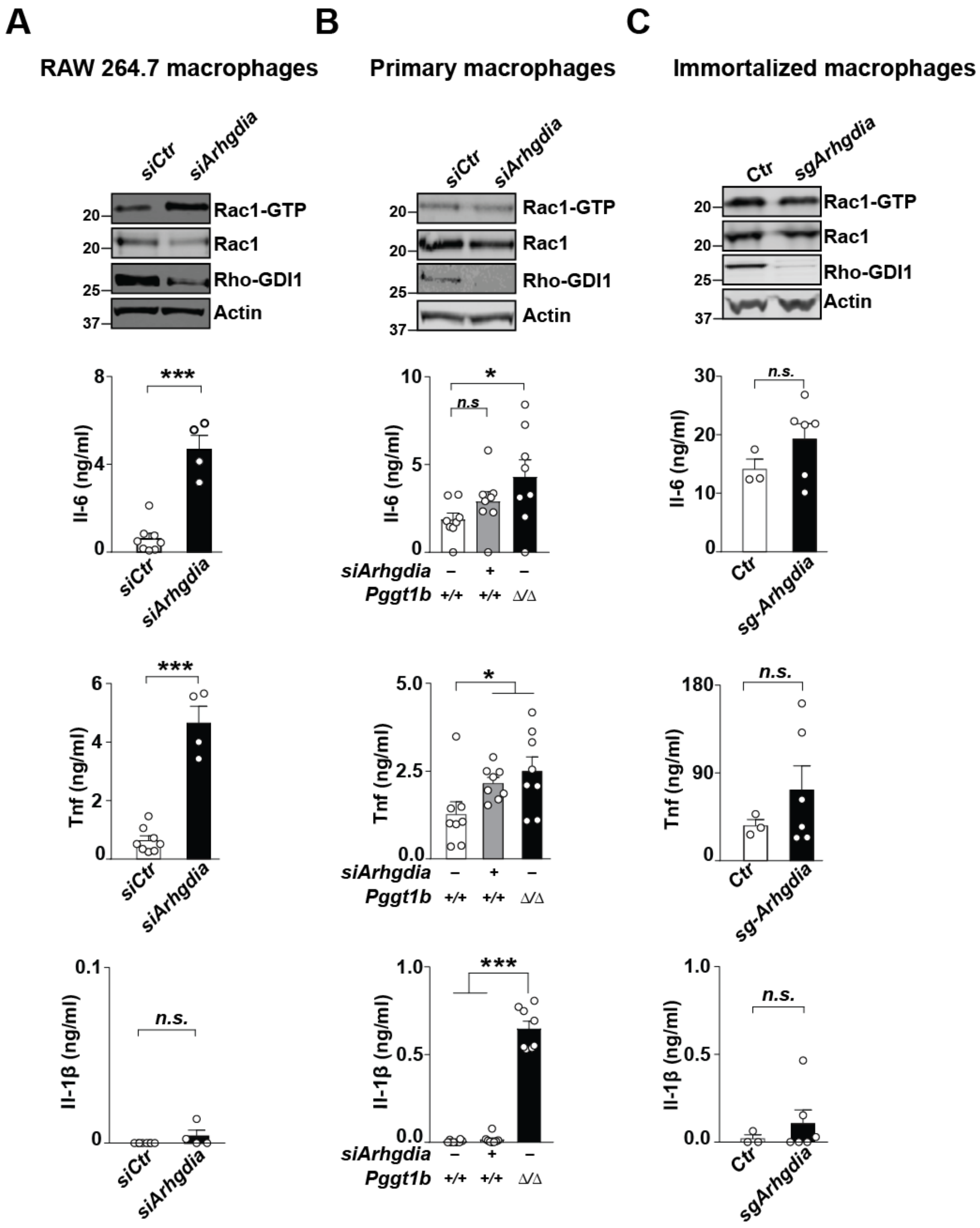

Supplementary Figure 4. Inactivation of Rho-GDI1 does not reproduce inflammatory phenotypes observed in GGTase-I knockout macrophages. (**A**) Upper panel, western blots showing steady-state levels of GTP-bound and total Rac1 and Rho-GDI1 in RAW 264.7 macrophages pre-incubated for 24 h with scrambled (*siCtr*) or *Arhgdia*-targeted siRNAs. Actin was the loading control. Lower panels, cytokine concentrations in macrophage medium, 8 hours after LPS stimulation. (**B**) Similar experiments as in panel **A** performed with primary BM macrophages. *Pggt1b*$^{\Delta/\Delta}$ macrophages were used as control in the cytokine analyses. (**C**) Similar experiments as in panels **A** and **B** performed with immortalized macrophages incubated with recombinant CAS9 and synthesized guideRNAs (sg) targeting exon 2 of *Arhgdia*. Error bars represent s.e.m. Significance between groups were calculated with two tailed Student's *t* test. *n.s*. not significant, * $P < 0.05$, *** $P < 0.001$.

Supplementary Figure 5

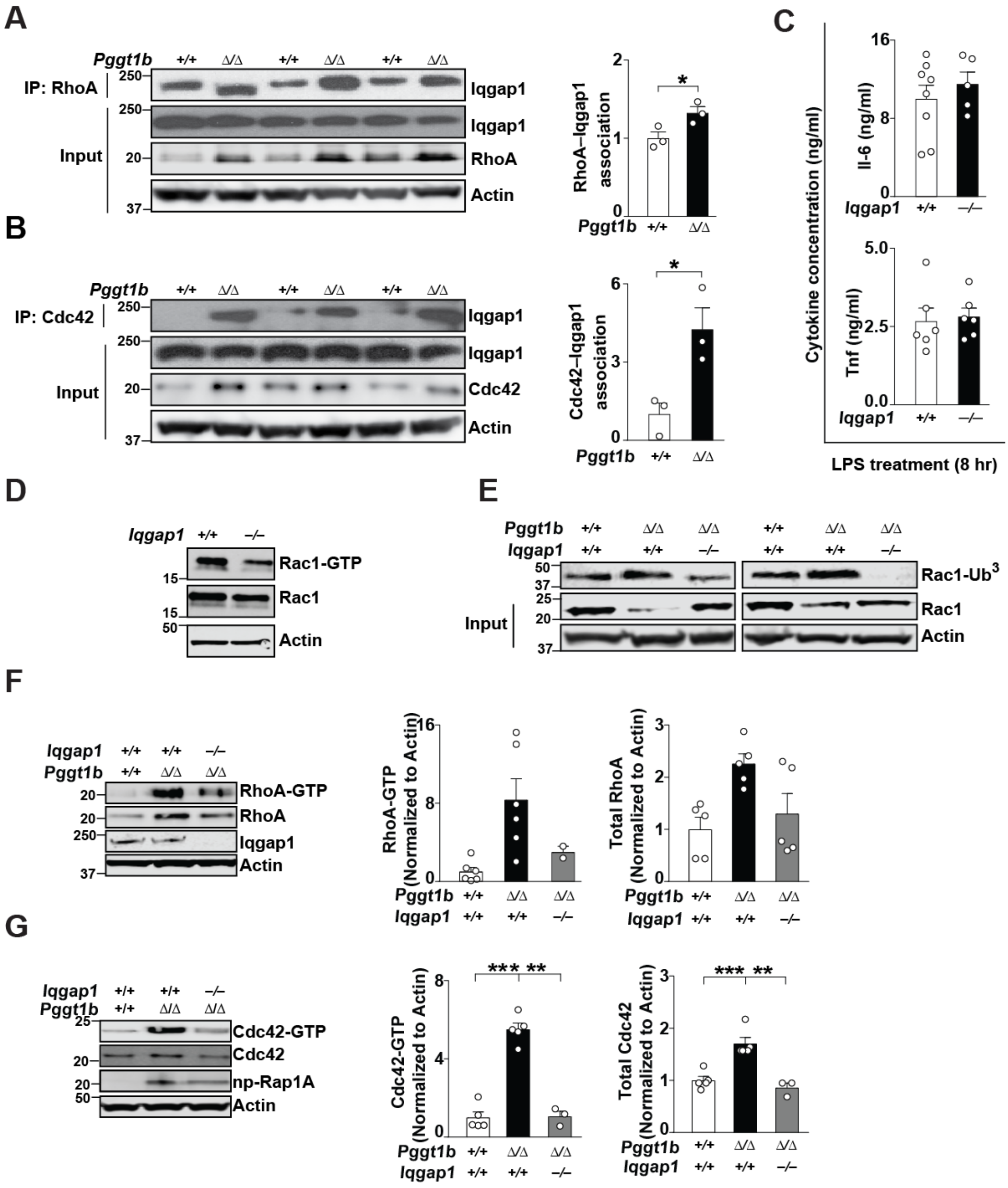

Supplementary Figure 5. Iqgap1 is functionally involved in the increased RhoA and Cdc42 activation and the increased Rac1 ubiquitination in GGTase-I-deficient macrophages. (**A, B**) Left panels, immunoprecipitation (IP) of RhoA (**A**) and Cdc42 (**B**) in BM macrophage lysates followed by western blots for Iqgap1. Direct western blots were performed on the same lysates (Input) to quantify total Iqgap1, RhoA, and Cdc42, and the loading control Actin. Right panels, levels of RhoA- (**A**) and Cdc42-bound (**B**) Iqgap1 were determined by densitometry of IP-western blots of BM macrophages from 3 mice/genotype. * $P < 0.05$. (**C**) Knockout of *Iqgap1* does not affect LPS-stimulated cytokine production in *Pggt1b*$^{+/+}$ macrophages. Concentration of Il-6 and Tnf in medium of BM macrophages isolated from *Iqgap*$^{–/–}$ and littermate control *Iqgap1*$^{+/+}$ mice (n = 2/genotype), 8 hours after LPS stimulation. (**D**) Western blots showing levels of GTP-bound Rac1 in lysates of BM macrophages isolated from *Pggt1b*$^{+/+}$ and *Iqgap1*$^{–/–}$ mice. (**E**) Knockout of *Iqgap1* reduces Rac1 ubiquitination in GGTase-I-deficient macrophages. Immunoprecipitation (IP) of Ubiquitin (Ub) followed by western blots for Rac1. Direct western blots were performed on the same lysates (Input) to quantify total levels of Rac1 and the loading control Actin. (**F, G**) Knockout of *Iqgap1* reduces GTP-bound and total RhoA and Cdc42. Left panels, western blots showing GTP-bound and total RhoA (**F**) and Cdc42 (**G**), and Iqgap1 in BM macrophages. Non-prenylated (np)-Rap1A was used as a marker of GGTase-I-deficient cells; Actin was the loading control. Right panels, levels of GTP-bound and total RhoA (**F**, n = 2–4/genotype) and Cdc42 (**G**, n = 3–5/genotype) in BM macrophages determined by densitometry. Error bars represent s.e.m. Significance between groups were calculated with two tailed Student's *t* test. *n.s.* not significant, * $P < 0.05$, ** $P < 0.01$, *** $P < 0.001$.

Supplementary Figure 6

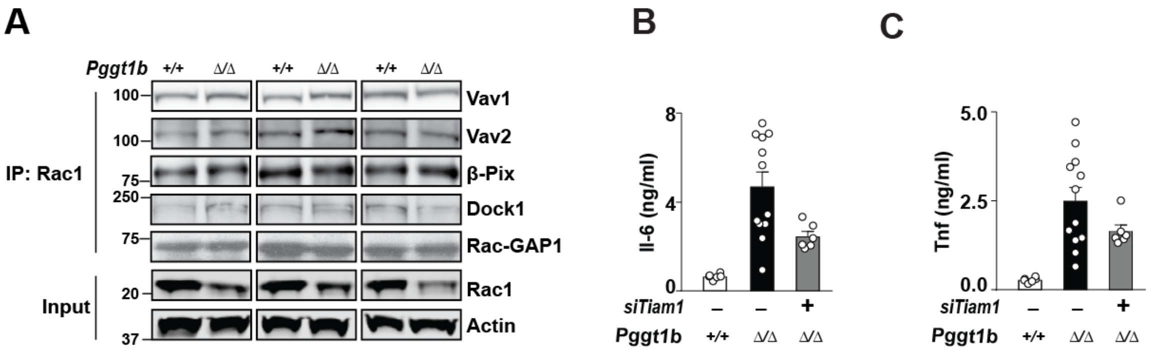

Supplementary Figure 6. GGTase-I deficiency does not affect the association between Rac1 and common GEFs and a GAP; and Tiam1 suppression reduces cytokine production in GGTase-I-deficient macrophages. (**A**) Immunoprecipitation (IP) of Rac1 in BM macrophage lysates followed by western blots for the guanine nucleotide exchange factors (GEFs) Vav1, Vav2, β-Pix, and Dock1; and for the GTPase-activating protein (GAP) Rac-GAP1. Direct western blots were performed on the same lysates (input) to quantify total Rac1 and Actin levels. (**B, C**) Tiam1 contributes to the increased cytokine production of GGTase-I-deficient macrophages. Concentrations of Il-6 (**B**) and Tnf (**C**), 8 hours after LPS stimulation, in medium of BM macrophages (*n* = 2/genotype) pre-incubated for 24 h with scrambled (–) or *Tiam1*-targeted (+) siRNAs. Error bars represent s.e.m.

Supplementary Figure 7

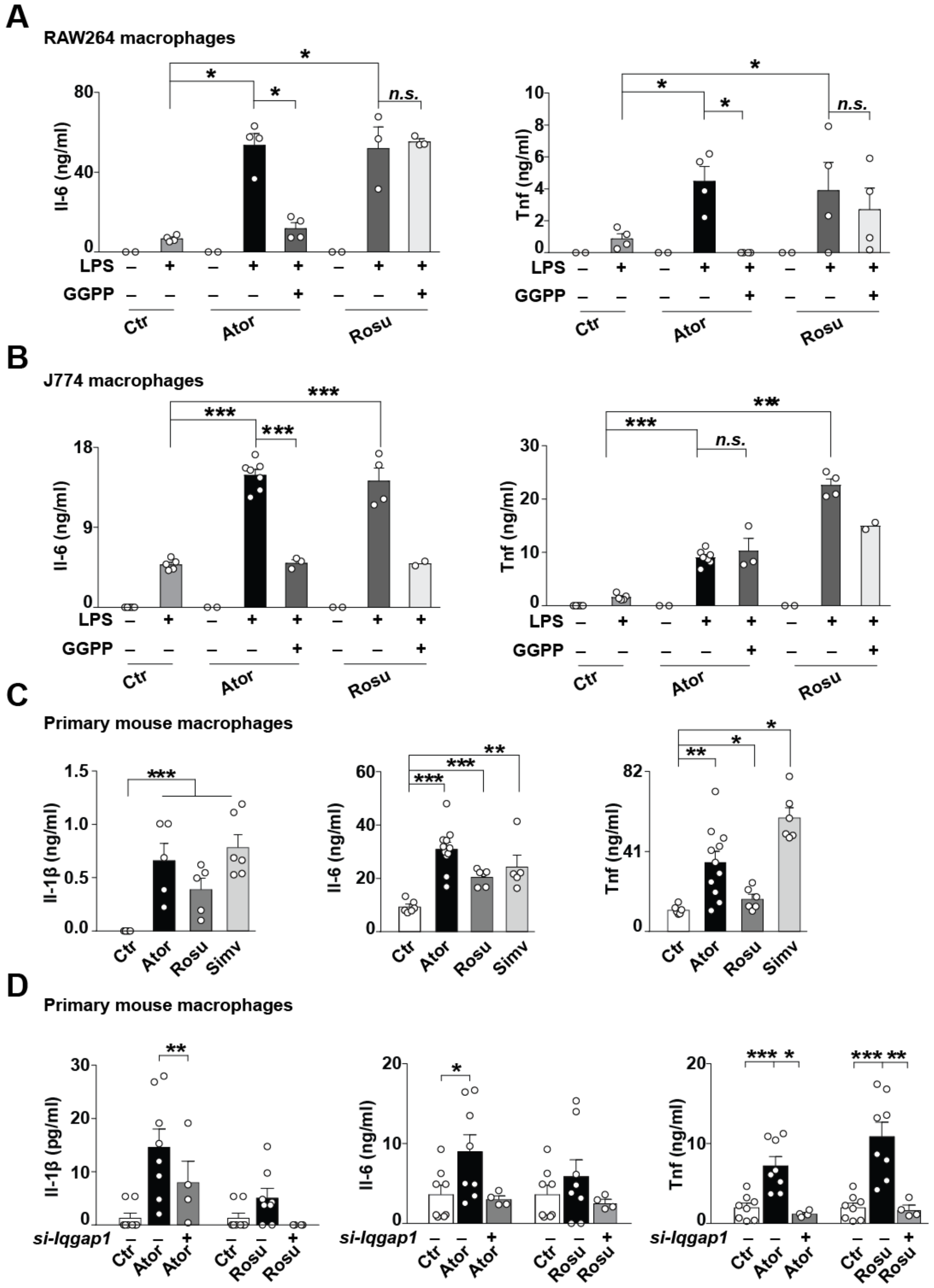

Supplementary Figure 7. Statins increase LPS-induced secretion of inflammatory cytokines in macrophages. (**A**) Il-6 and Tnf concentration, before and 8 hours after LPS stimulation, in medium of RAW 264.7 macrophages incubated for 18 days with Atorvastatin (5 µM) and Rosuvastatin (2.5 µM). GGPP (10 µg/ml) was added to the cells 3 days before LPS stimulation. (**B**) Similar experiment as in **A** performed with J774 macrophages. (**C**) Il-1β, Il-6, and Tnf concentration, 8 hours after LPS treatment, in medium of primary mouse BM macrophages (from n = 3 mice) incubated for 60 h with Atorvastatin (10 µM), Rosuvastatin (10 µM), and Simvastatin (5 µM). (**D**) Cytokine concentrations in medium of primary mouse macrophages incubdated with Atorvastatin (10 µM) and Rosuvastation (10 µM) for 60 h; scrambled or *Iqgap1*-targeting siRNAs were added 48 h before LPS stimulation. Error bars represent s.e.m. Significance between groups were calculated with two tailed Student's *t* test. *n.s.*, not significant, * $P < 0.05$, ** $P < 0.01$, *** $P < 0.001$.

Supplemental Figure 8

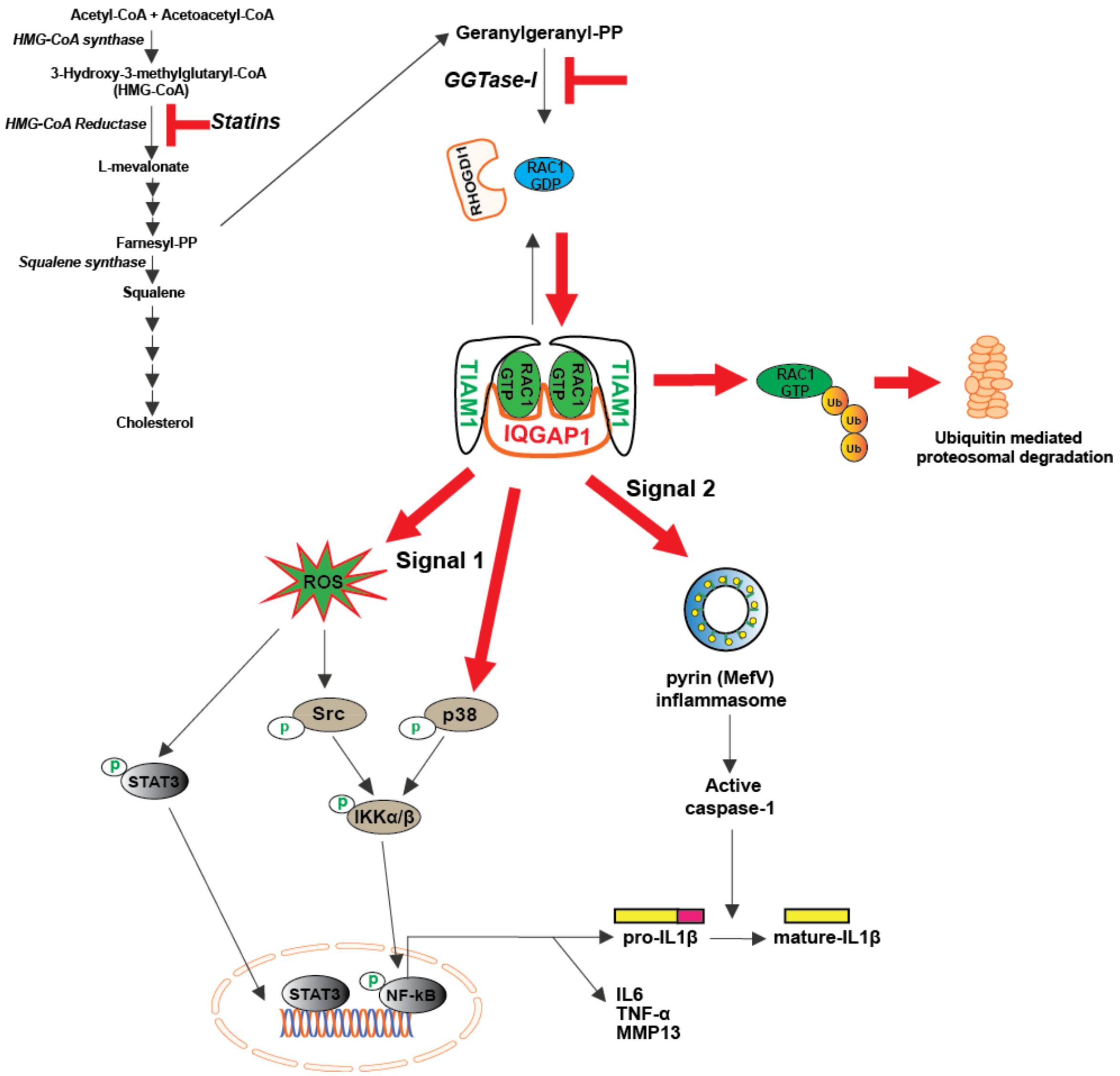

Supplementary Figure 8. Protein prenylation restrains innate immune responses by limiting Rac1 interactions with Iqgap1 and Tiam1. Rac1 and other Rho family proteins undergo posttranslational prenylation with the cholesterol biosynthetic intermediate geranylgeranyl-pyrophosphate (GGPP). Prenylation of Rho proteins can be blocked by inhibiting GGTase-I and by reducing the synthesis of GGPP, the latter of which occurs in cells from patients undergoing statin therapy and from patients with mevalonate kinase deficiency (MKD). In normal macrophages, prenylated Rac1 binds to Rho-GDI1 and has a low affinity for Iqgap1 and Tiam1 and thus exhibit low levels of GTP binding and activity. LPS stimulation activates moderate levels of Rac1 signaling and results in Il-6 and Tnf production; mature Il-1β is not produced due to the requirement for a second signal in the activation the inflammasome/caspase-1 complex. When prenylation is inhibited, non-prenylated Rac1 binds weakly to Rho-GDI1 and strongly to Iqgap1 and Tiam1 which facilitates GTP-loading and hyperactivates the protein. In this setting, LPS stimulation leads to high levels of signaling by ROS, Stat3, Src, and NF-κB and results in high levels of Il-6 and Tnf production; non-prenylated Rac1 also acts as a second signal in the production of active caspase-1 and maturation and secretion of Il-1β. *In vitro*, the increased cytokine production in the absence of GGTase-I depended on Rac1 and Iqgap1 and also on ROS generation and p38 activity; and *in vivo*, erosive arthritis development depended on Rac1 and Iqgap1.

Supplementary Table 1: List of primers used in this study

Gene	Primer
mIL1-β	Forward 5^1- CAACCAACAAGTGATATTCTCCATG -3^1<br>Reverse 5^1- GATCCACACTCTCCAGCTGCA-3^1
mCsf2	Forward 5^1-GCCATCAAAGAAGCCCTGAA-3^1<br>Reverse 5^1-GCGGGTCTGCACACATGTTA-3^1
mTnsf10	Forward 5^1-TCAGCACTTCAGGATGATGG-3^1<br>Reverse 5^1-CACCAGCTGTTTGGTTCTCA-3^1
mMmp12	Forward 5^1-AACTGGACAACTCAACTCTGGC-3^1<br>Reverse 5^1-CATCTCCTTGAATACCAGGTCC-3^1
mMmp13	Forward 5^1-TGTCATAACCATTCAGAGCC-3^1<br>Reverse 5^1-AGGTCTGGTCTGATGTGACACC-3^1
mCsf3	Forward 5^1-TGCTTAAGTCCCTGGAGCAA-3^1<br>Reverse 5^1-AGCTTGTAGGTGGCACACAA-3^1
mAdamts1	Forward 5^1-CTGGGCAAGAAATCTGATGA-3^1<br>Reverse 5^1-AAGCACAGCCACAGTTTATCA-3^1
mGapdh	Forward 5^1-TGCACCACCAACTGCTTAG-3^1<br>Reverse 5^1-GATGCAGGGATGATGTTC-3^1
