# Supplementary material for: Protein prenylation restrains innate immunity by inhibiting Rac1 effector interactions
Source: Nat Commun. 2019 Sep 4;10:3975. doi: 10.1038/s41467-019-11606-x (PMC6726657; doi:10.1038/s41467-019-11606-x)
Supplement: Supplementary file 3 — Reporting Summary [file 41467_2019_11606_MOESM3_ESM.pdf]

nature research

Corresponding author(s): Prof. Martin Bergo

Reporting Summary

Nature Research wishes to improve the reproducibility of the work that we publish. This form provides structure for consistency and transparency in reporting. For further information on Nature Research policies, see Authors & Referees and the Editorial Policy Checklist.

Statistical parameters

When statistical analyses are reported, confirm that the following items are present in the relevant location (e.g. figure legend, table legend, main text, or Methods section).

n/a	Confirmed	
☐	☒	The exact sample size (*n*) for each experimental group/condition, given as a discrete number and unit of measurement
☐	☒	An indication of whether measurements were taken from distinct samples or whether the same sample was measured repeatedly
☐	☒	The statistical test(s) used AND whether they are one- or two-sided<br>*Only common tests should be described solely by name; describe more complex techniques in the Methods section.*
☒	☐	A description of all covariates tested
☐	☒	A description of any assumptions or corrections, such as tests of normality and adjustment for multiple comparisons
☐	☒	A full description of the statistics including central tendency (e.g. means) or other basic estimates (e.g. regression coefficient) AND variation (e.g. standard deviation) or associated estimates of uncertainty (e.g. confidence intervals)
☐	☒	For null hypothesis testing, the test statistic (e.g. *F*, *t*, *r*) with confidence intervals, effect sizes, degrees of freedom and *P* value noted<br>*Give P values as exact values whenever suitable.*
☒	☐	For Bayesian analysis, information on the choice of priors and Markov chain Monte Carlo settings
☒	☐	For hierarchical and complex designs, identification of the appropriate level for tests and full reporting of outcomes
☒	☐	Estimates of effect sizes (e.g. Cohen's *d*, Pearson's *r*), indicating how they were calculated
☐	☒	Clearly defined error bars<br>*State explicitly what error bars represent (e.g. SD, SE, CI)*

Our web collection on statistics for biologists may be useful.

Software and code

Policy information about availability of computer code

Data collection	*Provide a description of all commercial, open source and custom code used to collect the data in this study, specifying the version used OR state that no software was used.*
Data analysis	Graphpad 8, ImageJ 2.0.0-rc, Image Studio Lite 4.0, Proteome Discoverer version 1.4, Zeiss Zen, BioTek Gen5, Microsoft Excel 2013, MatLab r2015a

For manuscripts utilizing custom algorithms or software that are central to the research but not yet described in published literature, software must be made available to editors/reviewers upon request. We strongly encourage code deposition in a community repository (e.g. GitHub). See the Nature Research guidelines for submitting code & software for further information.

Data

Policy information about availability of data

All manuscripts must include a data availability statement. This statement should provide the following information, where applicable:

- Accession codes, unique identifiers, or web links for publicly available datasets
- A list of figures that have associated raw data
- A description of any restrictions on data availability

The authors declare that the data supporting the findings of this study are available within the Article, supplementary information files and Source data, or are available upon reasonable requests to the authors.

Field-specific reporting

Please select the best fit for your research. If you are not sure, read the appropriate sections before making your selection.

☒ Life sciences ☐ Behavioural & social sciences ☐ Ecological, evolutionary & environmental sciences

For a reference copy of the document with all sections, see nature.com/authors/policies/ReportingSummary-flat.pdf

Life sciences study design

All studies must disclose on these points even when the disclosure is negative.

Sample size	No power calculation or sample size estimation was made as we know from experience that the effect sizes seen are large enough for simple N>3 group at all times.
Data exclusions	No data excluded from this study.
Replication	Most experiments were performed in triplicates or more, a few are only duplicates. The experiment in Fig. 5H was only performed once.
Randomization	Animals used for histological analysis and as sources for cells were only selected based on genotype, no other criterion was used.
Blinding	Synovitis and erosion of mouse joints were scored blindly by a collaborating group.

Reporting for specific materials, systems and methods

Materials & experimental systems

n/a	Involved in the study	
☐	☒	Unique biological materials
☐	☒	Antibodies
☐	☒	Eukaryotic cell lines
☒	☐	Palaeontology
☐	☒	Animals and other organisms
☒	☐	Human research participants

Methods

n/a	Involved in the study	
☒	☐	ChIP-seq
☒	☐	Flow cytometry
☒	☐	MRI-based neuroimaging

Unique biological materials

Policy information about availability of materials

Obtaining unique materials	We have described how to obtain all unique materials (mice) in the methods section.

Antibodies

Antibodies used	We have described all antibodies used in the study in the methods section.
Validation	We only acquired antibodies that were validated by manufacturers and that had been published previously.

Eukaryotic cell lines

Policy information about cell lines

Cell line source(s)	HEK293T cells, RAW 264.7 macrophages (ATCC) and J774 Macrophages (ATCC)
Authentication	Any non-unique cell line came directly from commercial sources that state that these cell lines are authentic. We did not do any inhouse identification post arrival of aliquots.
Mycoplasma contamination	All cell lines were tested for mycoplasma and were found negative.
Commonly misidentified lines (See ICLAC register)	*Name any commonly misidentified cell lines used in the study and provide a rationale for their use.*

Animals and other organisms

Policy information about studies involving animals; ARRIVE guidelines recommended for reporting animal research

Laboratory animals	All details about transgenic mice used in the study are described in the methods section. Both genders were used.
Wild animals	This study did not include wild animals.
Field-collected samples	No samples were collected from the field.
